# Re-bleed and Mortality Amongst Patients Following Initial Endoscopy for Upper Gastrointestinal Bleeding: A Single-Center Nigeria Study

**DOI:** 10.7759/cureus.12939

**Published:** 2021-01-27

**Authors:** Emeka Ray-Offor, Kalanne Opusunju

**Affiliations:** 1 Digestive Disease Unit, Oak Endoscopy Centre, Port Harcourt, NGA; 2 Department of Surgery, University of Port Harcourt Teaching Hospital, Port Harcourt, NGA

**Keywords:** upper gastrointestinal bleeding, endoscopy, outcome

## Abstract

Background and aim

Clinical and endoscopic parameters are predictive of patient outcome following acute upper gastrointestinal bleeding. The study aimed to investigate factors related to re-bleed and mortality following initial endoscopy among Nigerian patients with recent upper gastrointestinal bleeding (UGIB).

Methods

This is a cohort study of patients undergoing endoscopy for recent-onset UGIB at a referral endoscopy facility in Port Harcourt, Rivers State, Nigeria, from April 2014 to November 2020. Patients’ demographic and clinical data, American Society of Anesthesiologists (ASA) physical status, amount of blood transfusion, endoscopy results, and Rockall scores were retrieved from patients’ charts. The re-bleed and mortality rates were noted on follow-up by telephone. Statistical analysis was performed using SPSS version 20 (IMB Inc., Armonk, USA).

Results

A total of 560 patients had flexible video oesophagogastroduodenoscopy during the study period, and 46 (8.2%) of these were included in the study. Their age ranged from 28 years to 84 years (mean 58.6 ± 15.8 years) with 32 (69.6%) males and 14 (30.4%) females. Peptic ulcer disease (PUD) and gastritis/gastric erosions were the leading endoscopic diagnoses in 24 (52.2%) and 12 (26.1%) patients, respectively. Multiple comorbidities (p=0.021) and higher ASA score (mean 3.0; 95% confidence interval CI: 2.47-3.53; p=0.021) are associated with re-bleed, which was recorded in seven (15.2%) patients. Four (8.7%) cases of mortality were recorded in patients with a mean full Rockall score of 4.25 (95% CI: 1.52-6.97; p=0.021).

Conclusion

Re-bleed is more common in patients with multiple comorbidities, ASA score of three or more, and bleeding gastro-oesophageal varices at initial endoscopy. Mortality was significantly higher in patients with a full Rockall score of more than three.

## Introduction

Acute upper gastrointestinal bleeding (AUGIB) is a potential cause of a life-threatening emergency. Despite major advances in the last 40 years in managing AUGIB, morbidity and mortality have not changed significantly from reported rates of 10-12% and 5-10%, respectively [1, 2]. Common diseases associated with upper gastrointestinal bleeding (UGIB) include peptic ulcer disease, gastritis, and gastric erosion, gastro-oesophageal varices, oesophagitis, angiodysplasia, Mallory Weiss tear, and upper gastrointestinal malignancies. The modern methods used to determine upper gastrointestinal bleeding source include the radiological studies of triple vessel angiography or radionuclide studies, and endoscopy [3]. Upper gastrointestinal endoscopy (UGIE) is the procedure of choice in most patients with AUGIB, with a diagnostic accuracy of 80-95 percent [4]. For ease of memory, doctors managing patients with UGIB find the acronym BARE - bleeding, assessment, resuscitation, and endoscopy - useful [5]. Bleeding may stop spontaneously in the majority of patients without the need for any treatment besides hemodynamic support; however, there is the need for endoscopic evaluation to establish the etiology, effective treatment, and reduce the likelihood of re-bleed.

There are validated prognostic scoring systems that aid the stratification of patients with UGIB into low or high-risk patients [6]. These are predictive of patient outcome, assist decision making regarding the need for hospitalization in high-risk patients or a prompt, safe discharge in low-risk patients, and possibly influence the ideal time to perform endoscopy [7, 8]. Scoring systems used for UGIB have been grouped into three: endoscopic based only, endoscopic and clinical parameters combined and using clinical parameters only [7]. Among these scoring systems, the most used ones are the Glasgow‑Blatchford and Rockall scoring systems [8, 9]. The Glasgow-Blatchford score was developed to predict the need for intervention in UGIB, including transfusions, endoscopic therapy, and surgery but with the added need for laboratory data. The Rockall scoring system is simple to use, combining clinical information such as the patients’ age, the occurrence of shock assessed from systolic blood pressure records and pulse rate, presence and severity of comorbid conditions, and endoscopic stigmata of hemorrhage and diagnosis [10].

A search of literature from Nigeria, the most populous African country, shows a paucity of outcome studies from the South-south region of the country on acute upper gastrointestinal (GI) bleeding based on a validated endoscopy-based scoring system. This study aimed to investigate factors related to re-bleed and mortality following initial endoscopy among Nigerian patients with upper gastrointestinal bleeding in a metropolis of Nigeria.

## Materials and methods

This cohort study included consecutive patients with recent upper gastrointestinal bleeding referred for upper gastrointestinal endoscopy and performed at a referral endoscopy facility in Port Harcourt metropolis of Nigeria from April 2014 to October 2020. Pediatric patients (< 18years), patients presenting for endoscopy more than two weeks from the episode of bleeding, patients with gastric tumors, and those who could not be reached during follow-up were excluded from the study. Ethical clearance was obtained from the study center. The patients’ demographic and clinical data, including age, gender, American Society of Anesthesiologists (ASA) physical status grade, endoscopy results, blood transfusion, and Rockall scores, were retrieved from patients’ files in center records. Re-bleed and mortality rates were noted from follow-up telephone calls to patients or next of kin from in center records in the event of death.

Definitions

Hematemesis is defined as vomiting of blood, which may be frank red or appear as coffee grounds. Melena is a passage of dark, tarry stools. Hematochezia is a passage of frank blood per anus, alone or mixed with stools. Upper gastrointestinal bleeding is defined as any episode of vomiting blood or passing melena and/or haematochezia, whose source(s) of the bleeding is identified from the esophagus, stomach, or duodenum (proximal to the ligament of Treitz) at upper gastrointestinal tract endoscopy. For the study, recent upper gastrointestinal bleeding was presentation within two weeks of the episode. Shock is defined as systolic blood pressure below 100 mmHg. Re-bleed is defined as a separate episode of vomiting of fresh blood or melena or nasogastric evidence of new bleeding after endoscopy in the follow-up period, ranging from two weeks to five years. Mortality is a death related to UGIB within 60 days of presentation for endoscopy.

Endoscopy

A written and informed consent was obtained from every patient. The procedures were performed by a surgical endoscopist using Karl Storz (Karl Storz SE & Co, Tuttlingen, Germany) flexible video gastroscopes 13821/13801 PKS. A systematic examination of the upper digestive tract was done with the patient positioned in a left-lateral position preceded by local pharyngeal anesthesia using 10% lignocaine spray and sedation with an intravenous benzodiazepine. Oesophageal varices were graded as small, medium, and large based on the percentage of varix in the esophageal luminal radius. Gastric varices were graded using the Sarin’s classification: gastro-oesophageal varices (GOV)-1 and GOV-2 for the presence of prominent oesophageal varices with prominent gastric varices along the lesser curvature and the greater curvature, respectively; isolated gastric varices (IGV)-1 for fundal and cardia varices; and IGV-2 for varices in other parts of the stomach and duodenum [11]. Peptic ulcer disease was graded using the Forrest classification: an active spurting vessel hemorrhage as 1A or oozing hemorrhage - 1B; a non-bleeding visible vessel - IIA, adherent blood clot - IIB and hematin on ulcer base as IIC; and a clean base ulcer with no active bleeding as III [12]. Endoscopic hemostatic treatment was offered from a choice of adrenaline (one in 10,000 concentration), electrocoagulation, hemospray, clips, and band ligation as deemed suitable by the endoscopist. Patients were observed to be stable for a minimum of 30 minutes in the center after endoscopy, then discharged back to referring physician or transferred to a tertiary hospital. After the procedure, patients were followed up by telephone for recurrent bleeding and mortality over the post-procedure period.

Outcomes

The Rockall risk scoring system was used comprising clinical criteria (age, co-morbidity, presence of shock) and endoscopic (diagnosis, stigmata of recent hemorrhage) to stratify patients into low- and high-risk groups (Table 1) [10].

**Table 1:** Rockall scoring system

Variable	Score = 0	Score = 1	Score = 2	Score = 3
Age (years)	< 60	60-79	≥ 80	-
Comorbidity	-	-	Congestive heart failure, ischaemic heart disease, major disease	Renal failure, liver disease, metastatic disease
Shock	No shock	Pulse >100 beats per minute	Systolic blood pressure < 100mmHg	-
Source of bleeding	Mallory-Weiss tear	All other diagnoses, e.g., oesophagitis, gastritis, peptic ulcer disease, varices	Malignancy	-
Stigmata of recent bleed	-	-	Adherent clot or spurting vessel	-

The points were then added to produce a maximum pre-endoscopic Rockall score of seven and a maximum full Rockall score of 11. A score of less than three carries a good prognosis, but a total score of more than eight carries a high risk of mortality.

Statistical analysis

Statistical analysis of the data was performed using IBM SPSS Statistics for Windows, version 20.0 (IMB Inc., Armonk, USA). Mean, standard deviation, and percentages were summarized for continuous variables. The t-test was performed for numerical variables that were normally distributed. Multivariate logistic regression analysis was performed to identify independent predictors of re-bleed and mortality. Statistical significance was set at p<0.05.

## Results

Patient characteristics

Of 560 upper gastrointestinal endoscopies performed during the study period, 46 patients with upper gastrointestinal bleeding were included in the study. The age of patients ranged from 28 years to 84 years (mean 58.6 ± 15.8 years) with 32 (69.6%) males and 14 (30.4%) females. Nearly half of the patients (22/46) were 60 years and above (Table 2).

**Table 2:** Characterization of the study population ASA - American Society for Anesthesiologists physical status classification

Patient characteristics	Frequency (%)
Age group	
< 29 years	2 (4.3%)
30-39 years	6 (13.0%)
40-49 years	5 (10.9%)
50-59 years	11 (23.9%)
60-69 years	11 (23.9%)
70-79 years	6 (13.0%)
≥ 80 years	5 (10.9%)
Duration of presenting complaint	
≤ 2 days	17 (36.9%)
3-7 days	24 (52.2%)
8-14 days	5 (10.9%)
Comorbidity	
Hypertension	20/39 (51.3%)
Diabetes mellitus	12/39 (30.8%)
Cardiac failure	2/39 (5.1%)
Renal insufficiency	1/39 (2.6%)
Portal hypertension	3/39 (7.7%)
Advanced malignancy	1/39 (2.6%)
ASA	
II	21 (45.7%)
III	22 (47.8%)
IV	3 (6.5%)
Blood transfusion units	
0	15 (32.6%)
1-3 units	22 (47.8%)
≥ 4 units	9 (19.6%)

Haematemesis was the most frequent presenting complaint in 31 (67.3%) patients. There was a record of haematemesis alone in 14 (30.4%) and melena in 13 (28.3%), haematemesis and melena in 13 patients, and haematochezia with or without haematemesis in six (13.0%). Two or more comorbidities were recorded in patients with hypertension - 20 (51.3%), and diabetes mellitus - 12 (30.8%) the most frequent conditions. There was a positive history of non-steroidal anti-inflammatory drug (NSAID) abuse in six (13.0%) patients. Nearly half of patients - 22 (47.8%), received a transfusion of one to three units of blood before endoscopy.

Endoscopic diagnosis and treatment 

Peptic ulcer disease and gastritis/gastric erosions were the leading endoscopic diagnoses in 24 (52.2%) and 12 (26.1%) patients, respectively (Table 3).

**Table 3:** Endoscopic diagnosis in the study population

Pathology	Frequency (%)
Peptic ulcer disease	24 (52.2%)
Gastritis/gastric erosions	12 (26.1%)
Oesophageal/gastric varices	3 (6.5%)
Angiodysplasia	2 (4.3%)
No abnormality detected	2 (4.3%)
Mallory Weiss tear	1( 2.2%)
Duodenitis	1 (2.2%)
Obscure gastric bleeding	1 (2.2%)
Total	46 (100%)

All were clean base ulcers (Forrest III) but for three (12.5%) cases with a blood clot in ulcer floor (Forrest IIB) or haematin pigment (Forrest IIC). A sole case of moderate oesophageal varices was seen with a wale sign and two cases of gastric varices-GOV1 and IGV1 (Child-Pugh grade A). For endoscopic intervention, there were multiple rubber band ligation sessions for a case of oesophageal varices and injection sclerotherapy for gastric varices. A case of PUD received a 3 mls injection of adrenaline (one in 10,000 concentration). Also, there was a successful case of hemostasis using Hemospray for an obscure source of profuse gastric bleeding.

Outcomes

The majority of patients - 36/46 (78.3%) - had a full Rockall score of more than three (Table 4).

**Table 4:** Pre-endoscopic and full Rockall score among study patients NA - not applicable

Score	Pre-endoscopic Rockall score	%	Full Rockall score	%
0	13	28.3	1	2.2
1	19	14.3	13	28.3
2	10	21.7	13	28.3
3	2	4.3	9	19.6
4	1	2.2	4	8.7
5	0	0	3	6.5
6	1	2.2	2	4.3
7	0	0	1	2.2
>7	NA	NA	0	0
Total	46	100	46	100

There were seven (15.2%) cases of re-bleed: three out of 24 (12.5%) cases of PUD; two out of 12 (16.7%) cases of erosive gastritis; and one case out of three (33.3%) patients with oesophageal varices. There was no record of therapeutic surgery following the initial endoscopy. Re-bleed was more significant in patients with multiple comorbidities and higher ASA grade (p=0.021 and p=0.021, respectively). A higher mean full Rockall score was recorded among patients with re-bleed (Table 5).

**Table 5:** Comparing variables between re-bleed and no re-leed cases in the study population ASA - American Society for Anesthesiologists

Variable (n=7)	Re-bleed (mean)	95% Confidence Interval	No re-bleed (mean)	95% Confidence Interval	p-value
Age (years)	59.7	46.5-72.9	58.4	53.1-63.6	0.837
Number of comorbidities	1.57	0.67-2.47	0.74	0,48-1.01	0.021
ASA score	3.00	2.47-3.53	2.54	2.34-2.73	0.021
Full Rockall score	2.86	1.31-4.41	2.46	1.95-2.98	0.550
Blood pints transfused	1.86	0.61-3.10	1.89	1.29-2.51	0.947

Four (8.7%) mortalities were recorded; three (75%) out of these were in patients with full Rockall scores of four, five and six, respectively. A full Rockall score of more than three and an ASA score of three or more were significantly associated with mortality (p=0.027 and p=0.021, respectively), as seen in Table 6.

**Table 6:** Comparing variables between alive and mortality cases in the study population ASA - American Society for Anesthesiologists

Variables (n=4)	Mortality (mean)	95% Confidence Interval	Alive (mean)	95% Confidence Interval	p-value
Age (years)	65.5	43.35-85.65	57.9	52.91-62.90	0.327
Number of comorbidities	1.5	0.55-3.55	0.81	0.55-1.07	0.366
ASA score	3.25	2.45-4.05	2.55	2.36-2.73	0.027
Full Rockall score	4.25	1.53-6.97	2.36	1.89-2.82	0.021
Blood units transfused	1.75	0.64-4.14	1.8	1.33-2.48	0.857

From this study, age, sex, shock, number of comorbidities, or units of blood transfusion or NSAID abuse were not statistically significant as independent predictors of re-bleed or mortality.

## Discussion

In this study of upper gastrointestinal bleeding, patients in the fifth and sixth decades of life together presented with the highest frequency - 23.9% each; mean age of 58.6 years was recorded amongst patients with a male predominance. This trend is like a report on upper gastrointestinal bleeding from a tertiary health facility of another metropolis in South-south Nigeria; however, with reported a lower mean age of 51.5 years [13]. UGIB in the elderly carries a high mortality due to poor tolerance to blood loss from limited physiological reserve and frequent significant co-existing pathology [14]. It was observed that the mean age for cases of mortality was 65.6 years, and marginally more comorbidities were recorded in mortality cases (as indicated in Table 6). The re-bleed rate and mortality recorded from study patients were 15.2% and 8.7%, respectively. Marginally lower mortality of 5.9% is reported from a tertiary hospital in South-west Nigeria [15]. This difference is possibly related to the sample size.

Haematemesis was the most common presentation in patients with UGIB recorded from this study. This is dissimilar to global reports, including a report of melena in 93.4% of patients with UGIB from a similar Nigerian study [13, 15]. The presentation of dark tarry stool is associated with the presence of 50 mls or more of blood in the stomach, with other causes of the dark non-tarry stool as ingestion of iron tablets, bismuth, and green vegetables [16]. The reason for the trend in this study was possibly referral related as the alarming symptom of haematemesis more likely led to a prompt insistence by referring doctors for UGIE and acceptance by these patients as the majority of patients paid out-of-pocket for an endoscopy procedure. There were five (10.9%) cases only with health insurance coverage as the method of payment. Nine patients (19.6%) with UGIB received four or more pints of blood suggestive of massive UGIB, which is associated with hemodynamic instability. Three patients presented with systolic blood pressure <100 mmHg; however, eight patients had a pulse rate of >100 beats per minute. Massive upper GI bleeding from the duodenum may present as haematochezia hence the need for an upper and lower gastrointestinal endoscopy for evaluation of haematochezia [17]. A higher incidence of UGIB in the elderly is traced to increased use of NSAIDs [18]. A history of NSAID abuse was recorded in 13% of study patients.

Peptic ulcer disease was the most common etiology of UGIB diagnosed at endoscopy - 52.2%. This is similar but slightly higher than reports of 30.6% and 32.8% from two Nigerian studies [13, 15]. Similar reports are documented from Europe and North America [3, 6, 19]. In an earlier report from our center involving half of the present study population, gastritis/erosions (39%), peptic ulcer disease (13%), and oesophageal varices (4.3%), among others, were reported as common causes of UGIB diagnosed by endoscopy [20]. The trend of UGIB may have changed due to increased patient referrals. Chronic liver disease and splenic vein thrombosis are predisposing causes of life-threatening bleeding from oesophageal and gastric varices, as seen in three cases requiring transfusion of more than three units of blood. There are African and Middle Eastern reports of gastro-oesophageal varices as the leading cause of UGIB from Egypt, Malawi, Saudi Arabia, and Tanzania 31%, 43%, 47%, and 57%, respectively [21-24]. The suspected primary reason for these varices is the high endemicity of portal hypertension secondary to Schistosoma mansoni in some African countries [25]. An even higher rate of 67% for gastroesophageal varices related to AUGIB is reported from Punjab (India), with a high prevalence of alcoholic cirrhosis and hepatitis C [26].

UGIE is a veritable tool to diagnose the source of bleeding and offer treatment. Only 17 (36.9%) patients presented for endoscopy within 48 hours to onset of bleed, this possibly accounted for the low rate of stigmata of bleeding at endoscopy and a high rate of clean base ulcers and healing mucosal lesions. From the endoscopic hemostasis armamentarium available, injection sclerotherapy, variceal banding, and Hemospray were used in a few patients. In patients presenting with peptic ulcer bleeding, hemoglobin less than 10 grams, more than six units of blood transfusion, shock on admission, co-morbid disease, posterior wall duodenal ulcer, large ulcer size of >1 cm size, and Forrest lA predicted the failure of endoscopic therapy [27]. A higher number of comorbidities was statistically proven to be associated with re-bleed (p=0.021). The reported factors which are associated with higher mortality include esophageal varices as the bleeding source, the poor state of the liver reserve (Child's C), presence of comorbid diseases, age over 60 years, and a systolic blood pressure <90 mmHg on admission [24]. The Rockall score of more than three and ASA score of three or more in patients were statistically significant for mortality, but these factors were not identified as independent predictors of mortality from this study. In a higher-risk population of patients admitted directly to the intensive care with UGIB, predictors associated with 30-day mortality were ASA classification, comorbidity index, and duration of the hospital stay prior to upper gastrointestinal endoscopy [28]. A comparative study between Rockall scoring and Glasgow-Blatchford systems reports the former as superior in predicting a need for intervention as well as mortality while the latter being superior in predicting the need for blood transfusion [29]. Thus, the combination use of both risk stratification systems is recommended in clinical practice for patients with UGIB.

A limitation of this study is the sample size, which mirrors the referral culture in a teeming metropolis despite having limited endoscopy facilities. The duration to the presentation from the onset of bleeding was long in the majority of patients, increasing the chance of mucosal regeneration and possibly limiting the indication for endoscopic therapy. 

## Conclusions

Peptic ulcer disease, gastritis/gastric erosions, and gastro-oesophageal varices are leading causes of upper gastrointestinal bleeding, with endoscopy playing a key role in the management of these patients. However, there is a trend of long delays from onset of bleeding to presentation for endoscopic evaluation in our setting. Re-bleed is significant in patients with multiple comorbidities, ASA score of three or more, and bleeding gastro-oesophageal at initial endoscopy. Mortality of patients on the follow-up was significantly higher in patients with Rockall score of more than three hence the need for in-hospital expert multi-disciplinary team management of these group of patients.
